# Supplementary material for: COZOID: contact zone identifier for visual analysis of protein-protein interactions
Source: BMC Bioinformatics. 2018 Apr 6;19:125. doi: 10.1186/s12859-018-2113-6 (PMC5889581; doi:10.1186/s12859-018-2113-6)
Supplement: Supplementary file 2 — Software build. Executable binary file of the software tool. (ZIP 118,784 kb) [file 12859_2018_2113_MOESM2_ESM.zip › cozoid/platform/docs/testng-6.5.1-javadoc.zip!/org/testng/ConversionUtils.html]

ConversionUtils (TestNG)


---


|  |  |  |
| --- | --- | --- |
| **Overview** \| **Package** \| **Class** \| **Tree** \| **Deprecated** \| **Index** \| **Help** | |  |
| **PREV CLASS**   **NEXT CLASS** | **FRAMES**    **NO FRAMES**     **All Classes** |  |
| SUMMARY: NESTED \| FIELD \| CONSTR \| METHOD | DETAIL: FIELD \| CONSTR \| METHOD |  |


---


## org.testng Class ConversionUtils

```
java.lang.Object
  org.testng.ConversionUtils
```

---

``` public class ConversionUtils extends java.lang.Object ```

Helper methods used by the Eclipse plug-in when converting tests from JUnit.

**Author:**
:   Cedric Beust

---

**Constructor Summary**

`ConversionUtils()`


| **Method Summary** | |
| --- | --- |
| `static java.lang.Object[]` | `wrapDataProvider(java.lang.Class cls, java.util.Collection<java.lang.Object[]> data)`             Turns the output of a JUnit 4 @Parameters style data provider into one that is suitable for TestNG's @DataProvider. |

**Methods inherited from class java.lang.Object**

`clone, equals, finalize, getClass, hashCode, notify, notifyAll, toString, wait, wait, wait`

**Constructor Detail**

### ConversionUtils

```
public ConversionUtils()
```


**Method Detail**

### wrapDataProvider

```
public static java.lang.Object[] wrapDataProvider(java.lang.Class cls,
                                                  java.util.Collection<java.lang.Object[]> data)
```

:   Turns the output of a JUnit 4 @Parameters style data provider into
    one that is suitable for TestNG's @DataProvider.


---


|  |  |  |
| --- | --- | --- |
| **Overview** \| **Package** \| **Class** \| **Tree** \| **Deprecated** \| **Index** \| **Help** | |  |
| **PREV CLASS**   **NEXT CLASS** | **FRAMES**    **NO FRAMES**     **All Classes** |  |
| SUMMARY: NESTED \| FIELD \| CONSTR \| METHOD | DETAIL: FIELD \| CONSTR \| METHOD |  |


---
